# The Usefulness of Performing Biochemical Tests in the Saliva of Kickboxing Athletes in the Dynamic of Training

**DOI:** 10.1155/2019/2014347

**Published:** 2019-01-10

**Authors:** Oleksandr Anatolievich Volodchenko, Leonid Vladimirovich Podrigalo, Sergii Sidorovich Iermakov, Małgorzata Teresa Żychowska, Władysław Jagiełło

**Affiliations:** ^1^Department of Human Hygiene and Physiology, Kharkov State Academy of Physical Culture, Kharkov, Ukraine; ^2^Department of Tourism and Recreation, Gdansk University of Physical Education and Sport, Gdansk, Poland; ^3^Department of Life Sciences, Faculty of Physical Education, Gdansk University of Physical Education and Sport, Gdansk, Poland; ^4^Department of Sport, Gdansk University of Physical Education and Sport, Gdansk, Poland

## Abstract

The study aimed to determine the suitability of testing the saliva of kickboxing athletes to show changes in biochemical parameters in dynamic of training. 8 elite male athletes (mean age 17.29± 0.31 years, body mass 66.82± 3.46kg, with 5.62±0.96 years of training experience) participated in the study. Indicators of lipid peroxidation and glycolysis (the concentration of lactic acid and pyruvic acid) were defined before and after a training session. Significant increases in indicators of lipid peroxidation activity indicators and the concentration of lactic acid (4-fold) were observed; analysis of correlation matrices confirms the absence of expressed changes. At the same time, significant decreases in catalase (10-fold from 3.69 *μ*kat/L to 0.39 *μ*kat/L) and pyruvic acid (from 3.92 *μ*l/l to 0.55 *μ*l/l) were observed. Our results confirm the value of using saliva to determine training load in an individual. Moreover, the study provided information on the importance of indexes reflecting a correlation of various biochemical indicators to estimate the sufficiency of training loads. The ease of sampling and informational content of saliva are reasons to use such tests in monitoring athletes' functional state to prevent fatigue.

## 1. Introduction

Kickboxing is an increasingly popular and developing combat sport. As in any sport, it is important to develop and document a training program that will reliably and repeatedly develop the skills of individual athletes. In the current literature, there are reviews of the research devoted to the attributes of successful kickboxing athletes [1–3]. Podrigalo et al. [4] testified the importance of the cardiovascular system of athletes for the analysis of the capacity of aerobic adaptation potential. A goniometer was used in studies of kickboxers which correlated joint flexibility with mastery of the sport and athletes' success [5, 6].

Evaluation of changes in biochemical parameters is important for the prediction of success in sport. It is confirmed with results of many authors' research [7–9]; however, George et al. [10] emphasize the necessity of functional tests in addition to biochemical research. Other data [11, 12] confirm that biochemical tests allow the comparison and analysis of the efficacy of various exercise regimes. Perroni et al. [13] emphasized that differences in the effects of different modes of physical training could be revealed using biochemical tests. According to the results of Biersner et al. [13], the variability of biochemical parameters of team sports players is considered as one of the factors influencing effectiveness of the training. Moreover, Ouergui et al. [14] measuring lactate suggested that single kickboxing match is of sufficient intensity to stress the anaerobic metabolism.

Hammouda et al. [15] analyzed the responses of elite Tunisian football players to training: the authors confirmed their apparent correlation with physical qualities (in particular on endurance). Benedetti et al. [16] examined ultra-marathoners during competitions and recovery periods. In martial arts, biochemical changes due to a carbohydrate diet in Brazilian jiu jitsu athletes were analyzed [17], as was the impact of sodium bicarbonate during the recovery period in judo athletes [18]. Furthermore, Ouergui et al. [14] compared passive and active recovery on lactate concertation and influence on physical performance. The authors postulated that active recovery improved removal of lactate but did not improve subsequent performance. However, there are few studies of biochemical analysis of kickboxing athletes. Estimation of the training efficiency (i.e., development of physical fitness) of kickboxing athletes in competitions by means of biochemical tests was carried out by Zubac et al. [19].

Oxidative stress and antioxidant protection are important indicators of athletes' adaptation to training. Intense training loads in athletes could lead to overstrain and injury of the muscular system [8, 19–21], muscle hypoxia and an increase in the level of free radicals [22, 23]. Insufficient antioxidant (AO) protection could cause a decrease in an athlete's capacity to work and so reduce effectiveness. Some studies have shown that use of natural antioxidants decreased the level of oxidative stress in athletes and accelerated recovery [24]. It is known that oxidative stress is one of the most important limiting factors influencing athletic performance, by modulating physiological and biochemical reactions as well as gene expression associated with the cellular stress response [25, 26]. Therefore, determination of indicators of lipid peroxidation and enzymes involved in antioxidant protection is important to estimate athletes' training efficiency in different sports, such as tennis [27] and judo [28].

For this reason, the estimation of balance in the lipid peroxidation system- (LPS-) antioxidant system (AOS) is very important. The intensity of LPS reflects the level of oxidative stress in the organism. It allows prediction of the probability of exercise induced stress developing into muscle damage and pathology. AOS capacity illustrates the state of organism protection [29, 30]. In the considered context, this protection has to be estimated as an indicator of athletes' readiness. It can also be estimated as a capability of the highest achievements simultaneously with the optimum functional state. In our opinion, there are few studies in which lipid peroxidation and antioxidant protection power systems in kickboxing athletes were investigated; their change under the influence of training loads is still insufficiently studied and correlations between them are not traced enough.

Thus, the aim of this study was to evaluate the usefulness of performing biochemical tests in kickboxers' saliva in the training. We hypothesize that changes in lactate concentration, pyruvic acid, and antioxidative enzymes in saliva could be useful to determine training load.

## 2. Materials and Methods

### 2.1. Ethics Statement and Participants

This study was approved by the Bioethics Committee for Clinical Research and conducted according to the Declaration of Helsinki. All participants gave their written consent to research (protocol of the Commission on Bioethics of the Kharkov State Academy of Physical Culture No. 18) and were informed about the purpose and test procedures and about the possibility of withdrawal of consent at any time for any reason. The main criteria for the inclusion of athletes in the study were age (17-18 years old) and a high level of sportsmanship. This made it possible to estimate the dynamics of biochemical parameters of elite athletes under the influence of training loads. Eighteen athletes, aged 17.29 ± 0.31, mean body mass 66.82 ± 3.46 kg, and the training experience 5.62 ± 0.96 years, participated in the study.

### 2.2. Training

The athletes took part in a single session of 110-130-minute duration. The training consisted of the following: warm-up, general development exercises for all muscle groups and exercises stretching (30-35 minutes); main session block, kicking and striking techniques (40-45 minutes) and sparring (30-35 minutes); cool down, breathing and relaxation exercises (10-15 min). All of participants trained to the same session plan, did the same exercises, and used the same techniques.

The study was conducted in the precompetitive period. The training usually used the amount of physical activity, which is confirmed by the dynamics of pyruvic acid.

### 2.3. Sample Collection and Biochemical Analysis

Saliva was collected into special test tubes immediately before and 3-5 minutes after training, after tooth brushing. Samples were frozen and brought to laboratory within 12 hours. We did not use any means to stimulate salivation. Biochemical studies were conducted in a certified laboratory National Medical University in Kharkiv.

Products of lipid peroxidation, malondialdehyde (MDA) and diene conjugates (DC), indicators of the antioxidant system, catalase and superoxide dismutase (SOD) activity, and SH-group concentration were analyzed. These analyses were carried out according to standard methods: determination of diene conjugates was carried out spectrophotometrically; malondialdehyde was determined fluorometrically by reaction with thiobarbituric acid; catalase activity was determined spectrophotometrically with hydrogen peroxide substrate; superoxide dismutase activity was determined spectrophotometrically by the degree of inhibition of the nitro-blue tetrazolium reduction; and the concentration of SH-groups was determined spectrophotometrically with Ellman's reagent. Glycolysis indicators, concentration of lactic acid and pyruvic acid, were defined by means of “Olveks” reagents (Russia).

### 2.4. Statistical Analysis

The data were collected and arithmetic mean, standard deviation, and the error of arithmetic mean were calculated (Microsoft Word, EXCEL, 2010). The reliability of differences was estimated by means of parametric (Student's* t*-test) and nonparametric (sign test) indicators. Based on the results, correlation matrices were constructed, including Pearson's coefficient. The comparative analysis of the correlation matrices was carried out by means of indicators: specific weight of reliable and meaningful relationship, coefficient of labilization/synchronization (CL), and average correlation coefficient (ACC). The last two indicators were determined by means of special formulas:

(1) $$\begin{array}{c} CL=\left[\;\frac{n}{N\left(N-1\right)}\;\right]100\%, \end{array}$$

where n is the sum of all meaningful relationships formed by each parameter of the correlation structure; N is the total of parameters of the structure.

(2) $$\begin{array}{c} ACC=\frac{\Sigma rj}{n}, \end{array}$$

where Σrj is the sum of reliable relationships of correlation coefficients; n is number of meaningful relationships of the correlation.

The contribution of a separate criterion to the system was estimated according to the system-formation indicator (SI). This criterion reflects the number of connections formed by the studied indicator and their power. The indicator is expressed in conventional units (c.u.) and can be calculated using the following formula:

(3) $$\begin{array}{c} SI=\sum rj*n, \end{array}$$

where ∑rj is the sum of meaningful coefficients of correlation formed by this indicator; n is the number of meaningful relationships of this indicator of the structure.

## 3. Results

The essential changes of biochemical tested parameters were observed in saliva of athletes in the dynamics of training, confirmed by means of parametrical and nonparametric criteria. Changes in all tested parameters excluding pyruvic acid were significant; for malondialdehyde, diene conjugates, catalase (t = -7.12, p <0.05), superoxide dismutase (t = -4.83, p <0.05), and lactic acid, a significant increase was observed, while, for the -SH group (t = 6.02, p <0.05), it was a significant decrease. At the same time, no significant changes in pyruvic acid were observed (Table 1). The analysis of the sign test drawing similar conclusions. It was z = 0, p <0.01; z = 1, p <0.05 and z = 2, p <0.05, respectively. Changes in the concentration of pyruvic acid were less significant. By means of differences, it is not possible to distinguish between parametric and nonparametric indicators. The value of the Student* t*-test was t = -1.65, p> 0.05, sign test z = 8, p> 0.05.

Increase in the intensity of LPO was due to a significant increase in concentration of MDA and DC. It is proved by means of Student* t*-test, respectively: t = -21.87, p <0.001; t = -14.87, p < 0.001. The sign test was identical to both indicators, z = 0, p <0.01.

The Student* t*-test suggests the relationship between MDA/SH-groups; MDA/SOD and DC/SH-groups were the most informative. These results confirm their essential differences. Values of Student* t*-test were, respectively, as follows: -17.06, -2.43, and -30.21. All these ratios increase after the training session (Table 2).

Use of the sign test allows expansion of the conclusions to be drawn from the study. In the majority of athletes, there was an increase in MDA/catalase (z = 0, p <0.01), MDA/SH-groups (z = 0, p <0.01), DC/catalase (z = 1, p <0.05), and DC/SH-groups (z = 0, p<0.01). The value of the LA/PA index also significantly increased according to the sign test (z = 0, p <0.01).

The estimation of intensity of training is carried out according to the concentration of lactic and pyruvic acids. At the end of training the concentration of lactic acid increased 4-fold. The reliability of the observed differences is reflected in the parametric and nonparametric tests: Student* t*-test was t = -9.25, p <0.01; sign test z = 3, p <0.05.

Using correlation matrices also specified and increased the value of the obtained data. Key indicators of correlation structures are presented in Table 3.

Our results demonstrate that training loads did not lead to essential quantitative changes in correlation relationship. There was low variance in the observations both before and after training. Specific weight of significant correlations slightly increased (less than 5). Specific weight of significant relationships did not change in general. The level of the labilization/synchronization coefficient was low. The average correlation coefficient was also low.

It was found that quantitative indicators of correlation structures in the dynamics of training were rather similar. Therefore, it was interesting to analyze qualitative criteria. The system-formation indicators (SI) were calculated. It was established that, at the beginning of training, catalase (3.69) and pyruvic acid (3.92) have the maximum value of SI. At the end of training, indicators were significantly changed: the largest SI was determined for lactic acid (1.86) and MDA (1.83). SI of catalase decreased 10 times and was 0.39; SI of pyruvic acid was 0.55.

## 4. Discussion

The research described above allows analysis of the influence of training load on biochemical parameters. Similar research was conducted by Le Moal et al. [31]. Authors measured pro-/antioxidant status of professional football players and tried to link the pro-/antioxidant status with the training load. It was confirmed that athletes' oxidative and antioxidative status is altered according to training load. A similar design is used in research conducted by Kliszczewicz et al. [32]. The authors estimated the development of oxidative stress and antioxidant capacity of CrossFit athletes during intense training and the recovery period. This study confirmed that the intensity of exercise and the course of the recovery period influenced oxidative reactions. Changes in the LPO–AOS system of arm wrestling athletes during training and competition were studied by Podrigalo et al. [33]. Biochemical criteria for athletes with different levels of fitness illustrated the varying in biochemical adaptation to training.

Roh and So [34] in research conducted on obese and non-obese man during aerobic training confirmed the influence of physical exertion on the balance between oxidants and antioxidants in the body. Barranco-Ruiz et al. [35, 36] demonstrated similar results. In these studies, levels of superoxide dismutase, catalase, vitamins, and lipid peroxidation were analyzed to estimate the influence of physical training loads. The choice of parameters for the analysis was determined by their high informational content. Arsic et al. [37] used a similar set of criteria to monitor the condition of elite water polo athletes.

The choice of saliva as the research sample is determined by a range of factors. They include informational content, availability and ease of collection, and safety for participants. Similar results were described by Evans and Omaye [38]: estimating oxidative stress and the level of antioxidant protection through measurements in saliva was confirmed. Similar data were obtained by Grzesiak-Gasek et al. [39]: changes in saliva during training revealed changes in athlete metabolism. Drid et al. [40] analyzed sambo athletes' saliva to estimate the influence of physical exercises.

The observed increase in the intensity of lipid peroxidation (LPO) reflects adaptations under the stress of physical activity. This has been reported in many sports science publications. Arsic et al. [37] revealed that mechanisms of antioxidant protection adaptation are activated by physical activity and can be defined by the specific demands of the sport. Similar results were received by Perrea et al. [41]. The authors note that the volume of training and its nature have a significant effect on oxidative stress. Heaton et al. [42] determined that the state of the antioxidant system is an important performance enhancer in sport. The authors recommend an increase in the antioxidant potential to optimize recovery of athletes, even through supplementation.

The physiological antioxidant system (PAS) is a protective barrier against an increase in oxidative processes. Its enzymatic activity includes breaking down peroxide compounds and the neutralization of free radicals. The enzymes involved include superoxide dismutase and catalase, whose activity was examined in our work. Concentration of SH-group was studied because it provides insight into the factors of the enzyme system of glutathione peroxidase-glutathione reductase whose activity depends on the concentration of thiol-containing compound.

The results demonstrate the engagement of the antioxidant protection system and the increased use of thiol-containing compounds to neutralize free radicals. This is revealed by a decrease in the SH-group concentration. With a very high level of LPO, saturation and disruption of the AOS occurs. This is observed, for example, when patients are exposed to large doses of ionizing radiation [43]. However, physical exercise cannot lead to such a state; only adaptation stress is possible. This is confirmed by our results. In order to predict the status of oxidative stress in our athletes, we used the indices of the level of antioxidant protection, shown in Table 2.

Davies et al. [44] determined that intense training and an increase in oxidant formation lead to shifts in pro- and the antioxidants balance in the skeletal muscles, as well as development of oxidative stress, the phenomenon which is possibly the reason for muscular exhaustion and development of fatigue.

Ji [45] showed that long and regular physical training leads to an increase in the basal activity of some antioxidant enzymes (superoxide dismutases, glutathione peroxidase) and a decrease in others (catalase and glutathione reductase). Thivel et al. [46] confirmed existence of the influence of nutrition on the LPO–AOS system in rugby players. This decreased adverse effects of considerable physical loads.

Considerable training loads cause tension of buffer systems of organism, increase in the intensity of anaerobic oxidation, and a shift of рН with formation of metabolic acidosis. This results in an increase in the concentration of lactic acid in saliva. Similar results were observed by Šančić et al. [18] and Laskowski et al. [47]. Both sets of authors note the presence of considerable levels of lactic acid in martial art athletes during competition [48]. Laskowski et al. [47] used the lactic acid level to monitor the performance of judo athletes during the competition. The absence of significant changes in observed pyruvic acid concentration reflects a lack of metabolic changes. It confirms that the volume of physical activity does not exceed athletes' functional capacity.

Use of indexes to estimate the LPO-AOS system is determined by their simplicity and high informational content. Change of the index components allows defining the nature of adaptive shifts. The determined changes of indexes validate the hypothesis of adapting mechanisms by an increase in the intensity of LPO and a decrease in the potential of AOS. Increase in concentration of LPO is more significant. We conclude that limitation of oxidative processes becomes more difficult with increased antioxidants use.

The extensive informational content of the indexes (including the MDA definition) is confirmed by other researchers' results. For example, Shadab et al. [48] reported a significant increase in levels of MDA in serum after intensive training of game sports athletes.

The lactate/pyruvate index increased due to an increase in lactic acid. It reflects increased glycolysis; an increase in oxygen debt by means of increased rates of anaerobic oxidation. The stability of this index showed that the training volume was below functional limits of the athletes. The relation increases in our case that can be considered as work on the edge of possible (because of overload).

Reviewing correlation matrices allows estimating the dynamics of athletes' functional state. In the research of Rovnaya et al. [49], it was used to compare functional capabilities of synchronized swimmers. The analysis of indicators of correlation matrices confirmed adaptation during training. A comparison of correlation matrices of martial art athletes with different skill levels [50, 51] revealed a pattern; a higher level of LPS/AOS imbalance was seen in less experienced martial art athletes. The analysis of correlation matrices of biochemical indicators confirmed absence of significant shifts of adaptive mechanisms during a training program. The consistency of the observed criteria leads to the conclusion that the volume of training does not cause serious imbalances and instead corresponds to the athletes' functional skills; namely, the analysis of correlative structures once more supports the hypothesis that adaptation to stress stays within physiological limits.

Use of system-formation indicators in single combats athletes allows the estimation of the contribution of separate factors to the totality of the functional system. This was applied to different values of grasp strength in a pulsed mode for success of martial art athletes [52].

The dynamics of SI reflect changes in the body. At the beginning of the training session the condition at rest depends on the potential of AOS and aerobic oxidation. Therefore, the catalase and pyruvic acid had the maximum SI value. Physical activity leads to adaptation and oxidation intensification. It becomes a reason for an increase in contribution to the system of such indicators as lactic acid and LPO. However, the level is not significant, and increases in contribution are rather small, reflecting the absence of imbalance.

Therefore, the received results prove the available data concerning features of kickboxers' adaptive status. Results prove the significance of biochemical research for the prediction of success in this kind of sport. The used techniques of processing and information analysis allow differentiating more accurately the athletes' status in the dynamics of training to prevent development of overtraining. The study of the LPO–AOS system reflects the universal response of cell membranes to various influences, including physical exertion. The results obtained are informative and valid. Therefore, they can be used in monitoring the functional status of athletes. The dependence of the potential of AOS on diet allows us to recommend the modification of the diet as an ergogenic factor. However, this reaction is not specific and so should not be considered a “gold standard” of performance aid. Moreover, in this study we evaluated only biochemical markers from saliva and did not mark in the blood.

The choice of saliva as sample is due to its information content, accessibility and ease of collection, and relative biological safety. Our work serves as a basis for recommending the use of saliva to monitor the status of athletes. This allowed us to consider the goal set in the article as completed. According to literary data, the dynamics of indicators in the blood coincide with changes in saliva.

Practical recommendation: the obtained results allow us to recommend the analysis of the POL-AOC system in monitoring the functional status of kickboxing players as a tool for forecasting and readiness assessment. Available data proves the promise of using antioxidant diets to improve athletes' performance.

## 5. Conclusion

Study of kickboxers' reaction to training load has confirmed the value of the use of biochemical indicators in saliva. Activation of adaptable mechanisms is associated with imbalance in the LPO–AO protection system. The activity of LPO processes and the state of AOS as well as shifts in balance between them can be considered as indicators of the general state of the athlete's body. The specificity and sensitivity of these criteria allow estimation of regulation and homeostasis.

The values of the indexes reflecting a correlation of various biochemical indicators and studying the dynamics of correlation structures to estimate loads sufficiency have been confirmed. The informational content of saliva research is the basis for use of such studies in monitoring athletes' functional state.

## Figures and Tables

**Table 1:** Biochemical indicators of kickboxers' saliva in the dynamics of training.

**Indicator**	**Before training**	**After training**

**Malondialdehyde (MDA) (** ***μ*** **mol/L)**	4.57±0.25	9.81±0.25^∗^
**Diene conjugates (DC) (** ***μ*** **mol/L)**	24.46±0.31	37.79±0.53^∗^
**Catalase (CAT) (** ***μ*** **kat/L)**	41.71±0.35	47.85±0.79^∗^
**SH-groups (** ***μ*** **mol/L)**	2.08±0.16	0.85±0.13^∗^
**Superoxide dismutase (SOD)**	2.07±0.17	3.48±0.24^∗^
**Lactic acid (LA) (mM/L)**	0.48±0.08	1.95±0.14^∗^
**Pyruvic acid (PA) (** ***μ*** **mol/L)**	22.21±0.37	23.31±0.56

^*∗*^differences are significant (p<0.05).

The table presents the results of determining the mean values and their errors. These indicators are used to calculate the student's criterion. The difference was considered significant with a probability greater than 95% (p <0.05). The data was determined before and after the training; therefore the result groups were interrelated. Therefore, the character criterion was used to analyze the accuracy of the transfers. The difference was considered significant with a probability greater than 95% (p <0.05).

**Table 2:** Indexes of the antioxidant protection level in kickboxers in the dynamics of training.

**Index**	**Before training**	**After training**

**MDA/ CAT**	0.11±0.04	0.30±0.16
**MDA /SH-groups **	2.30±0.21	13.17±0.60^∗^
**MDA / SOD**	2.32±0.20	3.20±0.30^∗^
**DC / CAT**	0.59±0.06	1.15±0.31
**DC /SH-groups**	12.27±0.39	52.27±1.26^∗^
**DC / SOD**	12.55±0.41	12.70±0.69
**LA/PA **	0.02±0.02	0.09±0.04

*∗*: differences are significant (p<0.05).

**Table 3:** Indicators of correlation matrices of biochemical parameters of kickboxers before and after training.

**Measuring period**	**Specific weight of meaningful relationship (**%**)**	**Specific weight of reliable relationship (**%**)**	**Coefficient of labilization/synchronization (**%**)**	**Average correlation coefficient**

**Before training**	23.81±10.04	9.52±6.92	0.05	0.21
**After training**	28.57±10.65	9.52±6.92	0.06	0.18

## Data Availability

The data used to support the findings of this study are available from the corresponding author upon request.
